# Protein-Based Hydrogels: Promising Materials for Tissue Engineering

**DOI:** 10.3390/polym14050986

**Published:** 2022-02-28

**Authors:** Niyousha Davari, Negar Bakhtiary, Mehran Khajehmohammadi, Soulmaz Sarkari, Hamidreza Tolabi, Farnaz Ghorbani, Behafarid Ghalandari

**Affiliations:** 1 Department of Life Science Engineering, Faculty of New Sciences and Technologies, University of Tehran, Tehran 143951561, Iran; niyousha.davari@ut.ac.ir; 2 Burn Research Center, Iran University of Medical Sciences, Tehran 1449614535, Iran; negar.bakhtiary@modares.ac.ir; 3 Department of Biomaterials, Faculty of Interdisciplinary Science and Technology, Tarbiat Modares University, Tehran 14115114, Iran; 4 Department of Mechanical Engineering, Faculty of Engineering, Yazd University, Yazd 8174848351, Iran; mehran.khmm.mech@stu.yazd.ac.ir; 5 Medical Nanotechnology and Tissue Engineering Research Center, Yazd Reproductive Sciences Institute, Shahid Sadoughi University of Medical Sciences, Yazd 8916877391, Iran; 6 Department of Biomedical Engineering, Science and Research Branch, Islamic Azad University, Tehran 1477893855, Iran; ssarkari660@gmail.com; 7 New Technologies Research Center (NTRC), Amirkabir University of Technology, Tehran 158754413, Iran; tolabi@aut.ac.ir; 8 Department of Biomedical Engineering, Amirkabir University of Technology (Tehran Polytechnic), Tehran 158754413, Iran; 9 Institute of Biomaterials, Department of Material Science and Engineering, University of Erlangen-Nuremberg, Cauerstraße 6, 91058 Erlangen, Germany; 10 State Key Laboratory of Oncogenes and Related Genes, Institute for Personalized Medicine, School of Biomedical Engineering, Shanghai Jiao Tong University, Shanghai 200030, China

**Keywords:** tissue engineering, protein-based hydrogel, protein structures, unfolding, interaction

## Abstract

The successful design of a hydrogel for tissue engineering requires a profound understanding of its constituents’ structural and molecular properties, as well as the proper selection of components. If the engineered processes are in line with the procedures that natural materials undergo to achieve the best network structure necessary for the formation of the hydrogel with desired properties, the failure rate of tissue engineering projects will be significantly reduced. In this review, we examine the behavior of proteins as an essential and effective component of hydrogels, and describe the factors that can enhance the protein-based hydrogels’ structure. Furthermore, we outline the fabrication route of protein-based hydrogels from protein microstructure and the selection of appropriate materials according to recent research to growth factors, crucial members of the protein family, and their delivery approaches. Finally, the unmet needs and current challenges in developing the ideal biomaterials for protein-based hydrogels are discussed, and emerging strategies in this area are highlighted.

## 1. Introduction

Tissue engineering (TE) has significantly evolved toward compensating for the major drawbacks existing in medicine, and has been gaining increasing attention, as millions of people are suffering from failure or loss of organs and tissues annually. Of note, TE relies on the progress and evolution of suitable scaffolds for perfectly imitating the extracellular matrix (ECM); in this regard, scholars have been in a great struggle to find structures possessing the desired characteristics, and over the years, they have investigated and developed various structures, such as nanofibers [1], sponges [2], thin films [3], nanoparticles [4], composites [5], and hydrogels [6], with different properties and origins for the fabrication of scaffolds. Among these structures, hydrogels are one of the most ideal and promising candidates because of their biomimetic and tunable features, as well as their versatile fabrication methods [7,8,9].

Hydrogels, attractive biomaterials comprised of three-dimensional (3D), hydrophilic, cross-linked polymeric networks, are capable of absorbing and retaining large amounts of biological fluids [10,11]. As degradable matrices, they are considered perfect starting points for neo-tissue growth, and can make transplanting cells into the human body via a simple injection possible with minimal invasion [12]. Hydrogels’ characteristics can be tailor-made utilizing cross-linking methods, and they can be stimulated by environmental changes like pH, temperature, metabolite concentration, osmotic pressure, and via specific molecules such as glucose or antigens; therefore, the abovementioned outstanding features mean that these intriguing structures have an extensive range of applications, including as food additives, superabsorbents, wound dressings, biomedical implants, TE scaffolds, diagnostic devices, drug delivery carriers, and biosensors [4,13,14,15,16]. 

Proteins display remarkable structural and functional characteristics suitable for the manufacture of hydrogels, such as biocompatibility, biodegradability, abundance, and reduced ability to induce tissue inflammatory responses [17,18,19]. Besides, by employing physical, chemical, and enzymatic treatments, proteins that have innate benefits for hydrogel development can be turned into hydrogels [17,20,21]. Indeed, all proteins have the potential to be cross-linked, particularly through amine and carboxylic acid functional groups [20]. Protein aggregation into a gel network is the primary governing mechanism for the protein’s gelation process, known as physical cross-linking [22]. The formed network maintains water within its structure, and can be stabilized through non-covalent cross-links, such as hydrophobic, van der Waals, and electrostatic interactions, as well as hydrogen bonds [23,24,25]. Alternatively, chemical cross-linking stabilizes the gel network using various methods such as covalent cross-linking, chemical coupling, and click reactions. Hydrogels obtained from this technique possess improved stability, controllable degradation rate, and perfect mechanical properties in physiological circumstances. Regarding this issue, the cross-linker concentration is of high importance, since it can affect hydrogels’ mechanical features, release properties, and degradation rates [22,26,27]. 

Protein-based hydrogels (PBHs) offer comparable structural, mechanical, and chemical properties to the ECM. They can be readily processed under favorable conditions in order to be harmonized with living cells [17,28]. In addition, proteolytic enzymes can degrade PBHs; thus, the characteristics mentioned above make them ideal candidates in biomedical fields, including TE, drug, gene, and growth factors (GFs) delivery [29,30,31]. 

Although PBHs have been designed in great detail, a lack of clear understanding about how proteins interact within these systems still remains. This review gives an overview of PBHs, with a primary focus on proteins’ structures and interactions. Thereafter, the proteins’ various types, sources, and formation procedures of PBHs are thoroughly discussed. Furthermore, some of the applications of PBHs in the TE field are presented. Subsequently, hydrogel-based GFs delivery systems, their delivery methods, and critical parameters are investigated. Ultimately, current challenges and intriguing prospects of these hydrogels for TE research are mentioned.

## 2. Protein-Based Hydrogels

Protein is composed of amino acids connected together via the peptide bonds through a condensation reaction [32,33]. Of note, proteins’ physicochemical properties, mainly the mechanical characteristics, are challenging in the biomedical engineering field [34]. To overcome this obstacle, combining proteins with polymeric hydrogels with dynamic covalent bonds enables the fabrication of PBHs with proper structure, stability, strength, and various unique features, such as stimuli-responsiveness or self-healing properties [35,36,37,38]. By applying some modifications to the fabrication factors, the PBHs can display special properties matching the desired application area [39,40]. 

**Figure 1:**
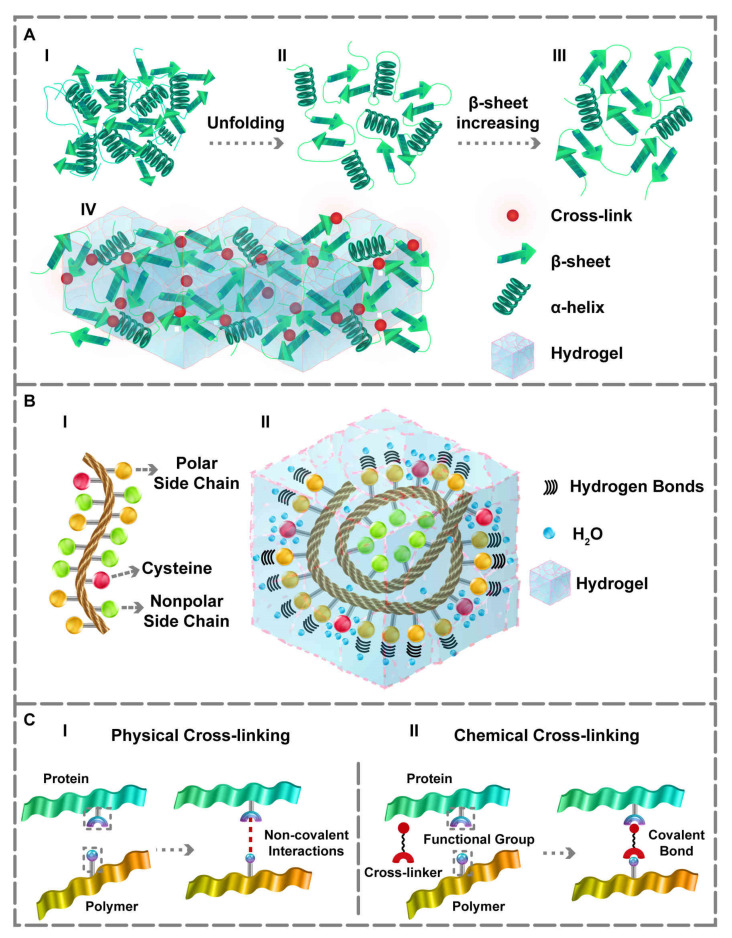
The protein’s unfolding and structural changes and its interaction within the hydrogel matrix. (**A**) (**I**,**II**) Conformational changes from the third to second structure. (**III**,**IV**) Increasing the β-sheet content which forms the desired gel matrix. (**B**) (**I**) Amino acids sequence. (**II**) Representation of hydrogen bonds within the hydrogel in the presence of polar side chains and the water-holding capacity of cysteine residue. The side chains of the polar amino acids make proper hydrogen bonds. Additionally, cysteine residue can form better hydrogels owing to the –SH group that assists water-holding capacity. (**C**) (**I**) Physical and (**II**) Chemical cross-linking approaches for obtaining PBHs gel matrix.

All proteins are composed of three main structures, and multichain proteins have a fourth structure. While the second structure describes details of the helix, β-sheet, turn, and loop contents, the third structure shows the protein’s 3D conformation [41]. All of the proteins’ properties, especially bioactivity, nature, and hydrophobicity, depend upon amino acid compositions at the primary level, leading to the secondary structures folding into the 3D tertiary structure [42]. Because proteins possess carboxyl and amino functional groups, converting them to hydrogels via physical, chemical, and enzymatic cross-linking approaches is possible. The most common and standard process in the protein gelation procedure is the protein unfolding and its accumulation into a gel matrix, as illustrated in Figure 1A(I,II) [22]. Conformational changes from the third to the second structure result in the rise of random coil content, with unlimited flexibility within the protein structure, which favors the structure for gelation [43,44]. In essence, the protein’s primary sequence is prepared for cross-linking and hydrogel formation through the protein unfolding. The major structural changes of protein for PBHs formation are due to the structural alteration from the third to second structure, and the changes in the secondary structure content. Owing to amino and carboxyl groups of proteins, a hydrophilic environment can be formed, leading to the emergence of high swelling property, an inherent feature of PBHs. Furthermore, proteins that contain cysteine can form better hydrogels compared to other proteins. It is because of the –SH group in the cysteine structure that assists the water-holding and absorption capacity (Figure 1B) [45]. Moreover, parameters like protein and polymer concentration, pH, and other hydrophilic functional groups impact the swelling ratio, which can be adjusted based on the application [45,46]. As an instance, Yan et al. [46] tuned the swelling ratio of the hydrogel’s interpenetrating polymeric network via regulating the concentrations of soy protein isolate and sugar beet pectin. Within another investigation, Joseph and co-workers [47] concluded that the incorporation of microparticles of fibrin could affect the swelling ratio and adhesion behavior of polyethylene glycol (PEG)-fibrinogen hydrogels. In similar research, Yan and colleagues [48] demonstrated that their corn fiber gum double-network hydrogel had a lower swelling rate in gastric fluid compared to the intestinal one, due to differences in the environmental pH.

PBHs’ gel matrix can also be achieved through covalent (chemical) and non-covalent (physical) cross-linking (Figure 1C). Fabricating PBHs via physical cross-linking approaches offers two significant advantages: (1) a simpler and non-cytotoxic method compared to the others, (2) maintaining most of the protein’s properties. In physical cross-linking, the interactions between the macromolecular chains are reinforced, enabling the manufacture of PBHs. Indeed, the weak intermolecular reactions leading to the formation of physically cross-linked hydrogels contain π-π stacking [49], electrostatic interactions [50,51], hydrogen bonding [52,53], and physical entanglement between molecules [34,54]. The prominent advantage of PBHs produced by physical cross-linking methods is the injectability and self-healing ability at the temperature of 25 °C. As proteins are naturally biocompatible substances, physical cross-linking techniques can preserve their biocompatibility to a sufficient extent and expand their application in the TE field [55]. Besides, in physical cross-linking, the proteins should be unfolded and moved back to the secondary structure so that the β-sheet content increment can form a gel matrix (Figure 1A(III, IV)). 

In this regard, through conformational unfolding, Hu et al. [56] enhanced the β-sheet content within silk protein employing ultrasound, followed by the physical cross-linking using hyaluronic acid. Pursuing a similar goal, Yan and co-workers added hyaluronic acid to silk protein solution in synergy with a solvent exchange and induced β-sheets structure into protein molecules, resulting in the direct assembling of a hydrogel with the same features as the silk protein [56]. Additionally, the protein concentration influences this type of physical cross-linking, meaning that in PBHs, the protein physically cross-links itself as the protein concentration goes up [57,58]. 

Because a single-matrix hydrogel often exhibits weak mechanical properties, hydrogels possessing single network wrapping or multiple networks superposition are alternative strategies for PBHs formation. Utilizing this method, Tang and colleagues [59] prepared a protein as the first matrix via protein misfolding strategy, in which applied heat to the protein caused its denaturation, aggregation, and gelation, and the polymer is the second one. Thereafter, they superimposed the matrices to produce a double-matrix PBH with significantly improved mechanical features and enhanced adhesion property. Furthermore, Xu et al. [53] developed a polyvinyl alcohol (PVA) solution in combination with bovine serum albumin (BSA) using a freeze-thawing strategy. Briefly, they fabricated the first abovementioned hydrogel matrix, and then tannic acid was cross-linked with PVA and BSA physically, in order to form a secondary hydrogel matrix using hydrophobic interactions and hydrogen bonds. It was revealed that the mechanical strength was increased in the protein-based double-matrix hydrogel constructed via superimposing the two assembled networks. Because of polymers’ and proteins’ long macromolecular chains, weak interactions, such as electrostatic interactions or hydrogen bonds, formed between these chains can be amplified. As a result, the complete denaturation of protein for forming hydrogel is not necessary and, to some extent, also unfavorable; in fact, the structural changes from the third to second structure or increased β-sheet content are sufficient in this process. Thermoplasticity, self-healing ability, recyclability, customizable remodeling, and reusability result from hydrogen bonds’ formation; thus, the residues can form hydrogen bonds with polymers, and the dynamic bonds are better formed alongside the polymers [52]. Concerning this subject, polar amino acids can create these hydrogen bonds (Figure 1B) [52,60]. In a novel study, Rahmani and co-workers manufactured hydrogels using proteins and alginate based on polar electrostatic reactions. Specifically, they modified the charge of the hydrogel matrix via the alginate microspheres’ cationic modification to prepare a hydrogel with a controlled release of protein-based drugs [50]. Moreover, Pacelli et al. [51] employed the same electrostatic interactions for physical cross-linking, and demonstrated that alginate could interact with gelatin to fabricate a coat of PBH based on interpenetrating the matrices of polymers.

The most important goal of the chemical cross-linking technique is the 3D network-structure PBH’s production through the formation of resistant and solid covalent bonds within its network [34,61]. The covalent cross-linking of target residues and particular chains on proteins [62,63,64], chemical coupling [19,65], and click reactions [66,67,68] are some of the chemical cross-linking approaches that have been reported. Enhanced stability, controllable degradation rate, and excellent mechanical properties in physiological conditions are achieved using this cross-linking method [69]. Besides, the origin of covalent bonds can influence the fabricated hydrogels’ structure. Proteins connect covalently in the presence of polymers, leading to the formation of hydrogels possessing new features. 

Several studies have employed chemical cross-linking methods to obtain PBHs with improved characteristics. In this regard, Kadumudi and colleagues created a mixture of silk proteins and redox graphene through non-covalent bonding, which led to a great decrease in proteins’ physicochemical properties [70]. Pursuing the purpose of covalent bonds formation, Hu et al. [39] chemically cross-linked modified graphene with resilin-like proteins and showed that several features such as adhesion, stretching, and sensitivity were enhanced. Additionally, Wang and co-workers utilized the same modification method and could form C=C bonds by introducing the vinyl group into gelatin, with the aim of producing gelatin methacrylate nanoparticles (GelMA NPs), which were the cross-linking core, and subsequently, ten wt.% acrylamide was added to GelMA NPs to fabricate macromolecular microsphere composite hydrogels [71]. It was revealed that the porous hydrogels had tunable pore sizes, improved elasticity, and enhanced compressive resistance. Furthermore, the effect of GelMA NPs dosage on the gel properties was determined: the higher the dose of GelMA NPs, the lower swelling ratio and the higher gels’ thermal stability and biocompatibility. In a recent study, Su and a group of scholars chemically cross-linked the proteins with chitosan to obtain covalent imide bonds and constructed hydrogels, encapsulating various particles using proteins charge [72]. 

Moreover, Chen et al. [73] covalently cross-linked PEG with T4 lysozyme mutant (T4M), in order to fabricate hydrogels for Zn^2+^ and Mg^2+^ release. The cross-linking was based on surface-located free amine functional groups of T4M and was performed employing non-toxic covalent cross-linkers for solidifying the hydrogel matrix. Besides, the free amine groups demonstrate a particular binding tendency to multivalent cations. Regarding the comparison between multi-block copolymers and single-block polymers, Phan and a group of researchers illustrated that a multi-block copolymer could display better properties than a single-block polymer by designing a three-block BSA-based hydrogel, and results showed that the cross-linking was improved in the multi-block copolymer because of the polymer’s structural complexity [74]. Within an innovative experiment, Huang and co-workers [57] embedded expanded silk networks into resilin blocks, and photo-chemically cross-linked the copolymers with the silk/resilin complex to obtain rubber-like hydrogels possessing the desired mechanical features. Increasing the silk-to-resilin ratio permitted the self-assembly of the resultant copolymers into fibers in a time-sensitive manner. Therefore, this event allowed the copolymer solutions’ controllable fibrillation at the supramolecular stage, which was obtained from the photo cross-linking of supported hydrogels, and demonstrated the effect of protein–polymer percentage on the fabrication mechanism of hydrogels. Furthermore, proteins in hydrogels can play a role as modifiers in achieving enhanced properties. Wang and co-workers prepared a PBH with anti-freezing, biocompatible, and tunable features, which was modified in two steps: (1) the addition of anti-freezing proteins associated with the natural fish, and (2) the employment of a chemical cross-linking method to obtain a polymeric hydrogel system [75]. Indeed, the structure of PBHs is modifiable, and polymers, owning cross-linking or modifying roles, can improve the hydrogel characteristics, since they form covalent bonds with proteins [76,77]. Polymers, as cross-linking agents, are capable of modifying the amine and carboxyl ends of proteins, resulting in fatigue-resistant hydrogels [77]. 

Enzymes, biocatalysts that can guide and assist the hydrogel production, offer considerable benefits, including performing cross-linking processes under mild conditions, possessing particular selectivity (chemo-, regio-, and enantioselectivity), and diminishing toxic cross-linkers [78]. As another cross-linking technique, enzymatic cross-linking was employed by Thi and co-workers in a novel experiment, in which an adhesive PBH containing gelatin was prepared via the dual-enzymatic cross-linking method using tyrosinase and horseradish peroxidase (HRP) [21]. Due to the easy reactions with nucleophiles, namely amines or thiols, and the conversion of gelatin’s functional phenol groups into o-quinone by tyrosinae, strong tissue adhesion was achieved. The dual-enzymatic cross-linking approach can produce hydrogels with remarkably improved adhesive strength. Chirilaet et al. [79] performed a self-cross-linking of silk fibroins via stimulating them by a HRP enzyme in the vicinity of hydrogen peroxide. The results confirmed the HRP-related advantages for the cross-linking procedure, and these privileges are significantly shorter gelation time, enhanced elasticity, and improved cytocompatibility. In another evaluation, Hou and colleagues fabricated a porous, cost-effective, simple, biofunctional PBH based on gelatin and enzymatic cross-linking by microbial transglutaminase (mTG) [80]. In this regard, mTG led to the emerging of gelatin microgels’ adhesive properties, and the formation of a bulk hydrogel with acceptable pore sizes suitable for cellular migration and proliferation. 

The biocompatibility of a hydrogel should be at a level that prevents environmental toxicity, and more importantly, inhibits the immune response of the host body [81]. In this regard, one of the components that can be used in hydrogels are proteins, natural materials similar to those of body components, which do not induce immune responses, leading to the failure of the hydrogel. Since proteins are the building blocks of the human body, they can be readily recognized by the body, and minimal immune reactions will occur when using PBHs; thus, the biocompatibility of PBHs is at a high level compared to other biomaterials. The PBHs have shown improved biocompatibility in the presence of proteins and polymers [38,56,71]. Herein, functional groups such as sulfhydryl and hydroxyl enhance the cross-linking process, and by regulating these groups’ concentration, the release rate, degradation rate, and mechanical properties can be adjusted [82]. In between, arginine and lysine residues have extra amine groups, and aspartic acid and glutamic residues possess additional carboxyl groups, increasing the need for cross-linking. Nonetheless, carbodiimides and aldehydes, the typical cross-linkers coupling the carboxylic acid-amine, are toxic and negatively affect cell survival and biocompatibility. To overcome this challenge, one of the cross-linkers that is more biocompatible than aldehyde is genipin, a small molecule obtained from gardenia fruit, that reacts with primary amines, and provides cross-link for collagen, silk, gelatin, and fibrin-based hydrogels just as well as carbodiimide [82]. In parallel, the results of Rafat et al. [83] examination on PEG combined with activated ester or aldehyde groups demonstrated that a considerable portion of PEG remained in the hydrogel and could modify its characteristics and reduce the amount of toxic cross-linker. 

### Protein Engineering for PBHs Formation 

Protein engineering is a cutting-edge technology that serves a significant role in biomedical applications [84]. This approach’s ability to create and enhance protein domains can be promising for making advanced functional hydrogels. These domains include coiled coil domains, leucine zipper, EF–hand domains (helix–loop–helix structural domain), and elastin-like polypeptides. Engineering the protein domains for achieving smart hydrogels in TE and delivering pharmaceutical agents such as anti-cancer drugs to a tumor site is an intriguing topic in the protein engineering field. In essence, the potential of well-engineered coiled coils to provide self-assembled hydrogels responsive to environmental stimuli makes coiled coils interesting options for controlled release and TE applications [85]. For example, a group of scientists fabricated a thermo-responsive hydrogel composed of a single coiled coil protein domain, which proved to be an ideal porous gel for small-molecule encapsulation [86]. Furthermore, engineered coiled coil domains are employed for improved targeting in a wide range of diseases, such as cancer and autoimmune illnesses [87,88,89]. For instance, a right-handed coiled coil (RHCC) with four identical alpha-helices was developed and used as a carrier system for platinum (IV), with the aim of enhancing the selectivity of the target tumors [90].

Various functional proteins have been engineered to be incorporated in the leucine zipper (LZ) protein backbone for the creation of LZ-based self-assembled hydrogels; nonetheless, the lack of the hydrogels’ stability makes them unfavorable candidates for TE applications. With the purpose of turning these hydrogels into suitable scaffolds for TE, a 3D self-assembling LZ hydrogel with tunable properties was produced, and it promoted cellular proliferation and attachment, and supported neo-vascularization [91,92]. Another example is employing the LZ domains or fusions to fabricate enzyme-responsive hydrogels; in this regard, a fusion consisting of a thermostable aldo-keto reductase, two alpha-helical leucine zippers, and randomly coiled domains was used to fabricate these functional hydrogels [93]. Engineering elastin-like peptides (ELPs) for the manufacture of ELP-based hydrogels is another instance in this field. The mechanical properties of the hydrogel can be tuned by creating blocks of lysine containing aliphatic and hydrophobic ELPs [94]. In an investigation conducted by Massodi et al. [95], a polypeptide carrier based on ELP was developed, in order to be employed for the chemotherapy at the tumor site. This study used L12, a peptide derivative of bovine lactoferrin, as an anti-cancer drug to induce apoptosis and necrosis in cancer cells at the tumor region.

Recently, a bilayer protein-based shape memory/morphing hydrogel with high adjustability and reversible deformation was optimized via the structural sequence [96]. Moreover, the interaction between self-assembled nanofibers leading to the hydrogel formation was improved with the fusion protein ULD (ubiquitin-like domain)-TIP-1 (Tax-interacting protein-1) by Zhang and a group of researchers [97]. They revealed that the final hydrogels’ mechanical properties depended on the kind of peptides that were bound to the TIP-1 protein via different binding affinities. Overall, all of the abilities that protein engineering demonstrates, including transforming the PBHs to advanced structures in TE and drug delivery fields, make it a qualified approach for facing monumental challenges in biomedical research.

## 3. TE-Related Applications of PBHs 

**Figure 2:**
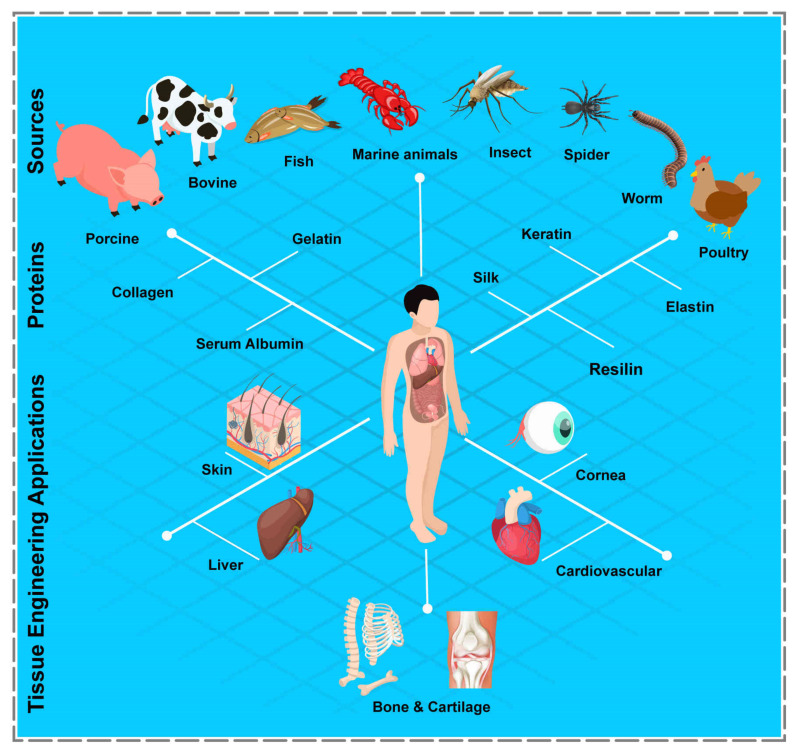
Schematic illustration of various common proteins, their sources, and PBHs’ TE-related applications. The figure was prepared based on the abundance of proteins.

Many scholars have employed PBHs for various tissues regeneration throughout the years (Figure 2). Utilizing PBHs allows us to include sequences that promote cellular attachment to the substrate and overall cell development. In essence, the structural design, unique biological function, and stimuli-responsiveness of PBHs’ components make them particularly desirable. Herein, advances in the field of PBHs, including hydrogel types, components, and their effects, with a prime focus on TE applications of PBHs, are presented. Table 1 summarizes the investigations conducted in this area.

### 3.1. Collagen-Based Hydrogels 

Collagen, the most abundant fibrous protein, is found within hard and soft tissues, such as connective tissue [126], skin [127,128,129], tendon [130], cornea [131], and cartilage [132,133,134,135], and is a crucial constituent of the ECM [136]. In this regard, collagen derived from the bovine [137], porcine [138], fish [139], marine sponge [140], shellfish [141], and jellyfish [142] is largely explored, so as to be utilized as a biocompatible material in various fields [143].

Collagen hydrogels, thermo-reversible, and physically formed hydrogels, exhibit poor physicomechanical features compared to the covalently cross-linked ones generated via glutaraldehyde or diphenyl phosphorylate azide [144,145]. Furthermore, gelation conditions significantly influence the hydrogel formation; to be more specific, parameters such as temperature and pH can be finely adjusted to tune the features of the collagen hydrogel’s fibrous matrix [146]. Concerning this subject, collagen-based hydrogels fabricated at lower temperatures demonstrate larger pore diameters, as well as enhanced osteoblast-like cell responsiveness [147], while those formed at a higher temperature (e.g., 37 °C) promote fibrillogenesis and illustrate smaller pore diameters [148]. 

The chemical functionalization of collagen throughout the extraction process removes its natural bonding; thus, biomaterials containing collagen require inter-and intra-molecular cross-linking, with the goal of improving their mechanical characteristics [149,150]. In particular, cross-linkers such as glutaraldehyde [151], gamma radiation [152], N-hydroxy succinimide [153], carbodiimide [154], formaldehyde [155], hexamethylene diisocyanate [156], sodium tripolyphosphate [157], genipin [158], transglutaminase [159], dialdehyde [160], and sugar-based [161] ones, are utilized to augment the collagen hydrogels’ stability that is necessary for preventing the quick enzymatic degradation [162]. Hence, the collagen-based hydrogel is recognized as a promising choice for TE applications.

Several studies have employed collagen-based hydrogels due to their desirable properties. Accordingly, Lee et al. [98] proposed a method in which the 3D bioprinting of collagen was possible via the freeform reversible embedding of suspended hydrogels (FRESH), in order to manufacture human heart components in different sizes. Adjusting pH-driven gelation resulted in reaching 20 µm fiber resolution, obtaining a porous structure that permitted rapid mouse myoblast cells’ (C2C12) infiltration and micro-vascularization, as well as achieving mechanical strength for the perfusion and production of tri-leaflet valves and multi-scale vasculature. Conducting further tests, they revealed that FRESH printed hearts could precisely reproduce the anatomical structure that was specific for each patient. Furthermore, cardiac ventricles printed alongside cardiomyocytes under ultra-violet (UV) exposure displayed contractions in sync with each other, and wall thickening of roughly 14% throughout peak systole, indicating the potential of this novel hydrogel for heart valves regeneration. 

Collagen fibrils that form a lattice pattern within the cornea structure serve a significant role in the cornea’s transparency. Inspired by this matter, Kim and co-workers [99] created a transplantable clear cornea consisting of an integrated structure of collagen fibrils. The structure was fabricated employing the 3D-printing process, via inducing shear stress to the hydrogel bioink composed of corneal stroma-derived decellularized ECM. Initially, the printed hydrogel-based complex was incubated for 30 min to maintain its mechanical stability. The printed hydrogel-based structure mimicked the cornea’s native macrostructure, and showed enhanced human turbinate-derived mesenchymal stem cells alignment capabilities, which indicated the collagen fibrils’ structural organization in the cornea. After the four-week implantation in ten healthy New Zealand white rabbits, the remodeled collagen fibrils produced a lattice pattern analogous to the natural cornea structure.

In another evaluation, Yang and colleagues [100] examined three distinct hydrogel compositions, including alginate, alginate-agarose, and collagen-alginate. The results revealed that the maximum chondrocyte growth was obtained in the collagen-alginate samples with increases of 29.96%, 135.16%, and 233.97%, compared to the alginate samples after three, seven, and fourteen days of culture, respectively. Additionally, the expression of certain cartilage gene markers by cells cultivated on the collagen-alginate hydrogels was much higher than other groups, and the formation of glycosaminoglycans was observed after two weeks of cultivation. Besides, the chondrocytes phenotype was effectively preserved when cells were placed into the collagen-alginate hydrogel.

**Figure 3:**
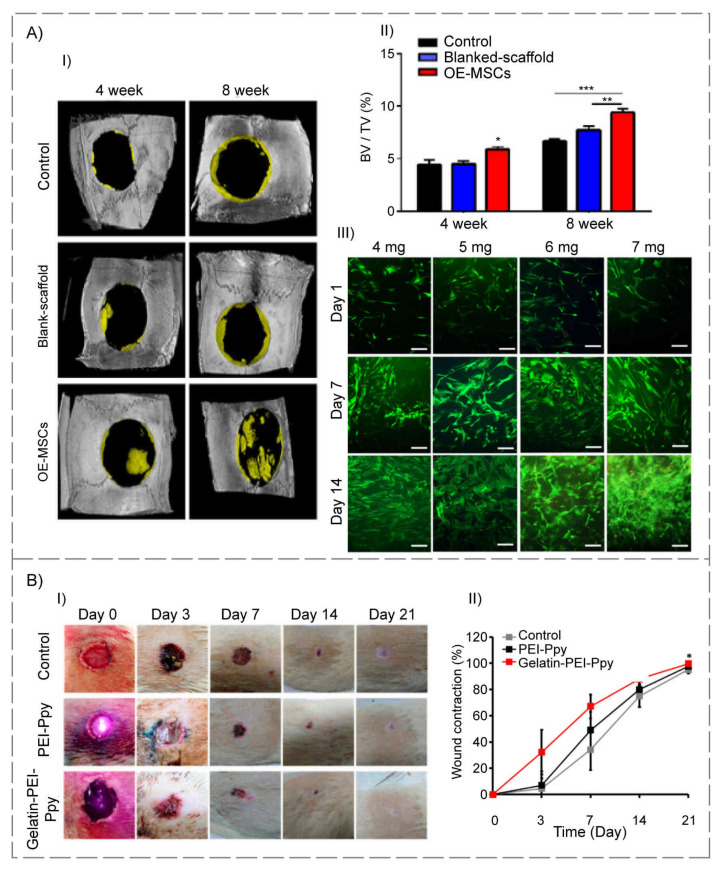
(**A**) Schematic of bone development and evaluation of human OE-MSCs’ viability that was encapsulated into different concentrations of collagen hydrogels for 14 days. (**I**) Representative of micro-CT images related to rat calvarial defects treated with or without OE-MSCs after 4 and 8 weeks. (**II**) In rat calvarial defect models, summarized data showed new bone tissue volume/total defect volume (BV/TV) for newly developed bone tissue. (**III**) Live/dead fluorescence images of OE-MSCs cultured on collagen hydrogels; the green color shows the living cells, and the red color indicates the dead cells (scale bar = 100 µm) (reproduced content is open access) [101]. (**B**) (**I**) Periodical wound healing evaluation (full-thickness wound in the Wistar rat model): macroscopic images of the wound site and wound area of the control and two experimental groups at different time points (day (d) 0, 3, 7, 14, and 21) (n = 3). (**II**) Wound contraction (%) at various stages of wound healing and complete wound closure from day 0 to day 21 (n = 3) (*p* < 0.05) (reproduced content is open access) [104].

Cell transplantation using polymeric carriers has recently received a lot of attention in the TE field. In this regard, Simorgh et al. [101] loaded human olfactoryecto mesenchymal stem cells (OE-MSC) in the cross-linked collagen-based hydrogels (incubated at 37 °C) and evaluated their osteogenic capacity, in vitro and in vivo. Concerning the subject that type I collagen is available in four distinct concentration levels (4, 5, 6, and 7 mg/mL), OE-MSCs were encapsulated in the optimal concentration of collagen-based hydrogel, and were then injected into rat calvarial lesions. Following four and eight weeks post-transplantation, bone samples were collected and analyzed. All of the hydrogel scaffolds were found to have higher porosity and bioactivity. The collagen-based hydrogel with a 7 mg/mL concentration had superior mechanical characteristics compared to the native bone. Additionally, real-time PCR and alizarin red S techniques verified that the structure with the same concentration displayed significant osteogenic differentiation. Furthermore, the in vivo treatment of defects with cell-loaded collagen-based hydrogels resulted in substantial bone repair. As a result, the collagen-based hydrogels that were viable, biodegradable, and biocompatible carriers can be employed to treat clinical bone defects (Figure 3A).

### 3.2. Gelatin-Based Hydrogels

Gelatin, a hydrophilic biopolymer derived from denatured collagen, has been widely employed in TE applications, due to its biocompatibility, non-immunogenicity, high carbon content, low cost, great availability, biodegradability, etc. [163,164]. Furthermore, it is readily soluble and displays amphoteric behavior at 37 °C [165]. For instance, due to their excellent fluid absorption capacity, gelatin-based hydrogels can be used as wound dressings [166]. The differing isoelectric point (pI) values of cationic and anionic gelatin (pI equal to pH 7–9 for type A and pH 4.7–5.1 for type B) should be considered when gelatin-based formulas are developed, since pI can affect the retention of gelatin’s active components [167]. Because of these characteristics, gelatin-based hydrogels are extensively used to produce contact lenses, TE scaffolds, and drug delivery systems [168,169].

The relatively poor thermomechanical stability is one of gelatin hydrogel’s drawbacks, but chemical functionalization and modification increase the durability of these hydrogels, and make them suitable for long-term biological applications [170]. Of note, gelatin’s functional groups, including primary amine, carboxyl, and hydroxyl, enable its modification with various cross-linkers and therapeutic agents, making it an ideal candidate for tissue regeneration [166]. The hydrophobic amino acids of gelatin, such as tyrosine and proline, can be cross-linked using glutaraldehyde [171], diisocyanates [172], carbodiimides [173], and genipin [174]. Additionally, being thermo-responsive, gelatin can exhibit a reversible sol-gel transition property; indeed, the conversion from solution to gel occurs whenever the temperature drops, and this alteration can be reversed by raising the temperature of the combination to the physiological temperature.

Nichol and co-workers [102] investigated the gelatin-based hydrogel’s integration with methacrylate in microscale form, in order to cultivate human umbilical vein endothelial cells (HUVEC) on micro-patterned GelMA hydrogels. This hydrogel was cross-linked under UV exposure in the presence of 0.5% *w*/*v* Irgacure 2959 as a photoinitiator. It was illustrated that cells rapidly adhered, proliferated, interacted, and migrated both in 2D and 3D cultures employing GelMA hydrogels. Hence, these findings demonstrated that GelMA hydrogels might effectively produce cell-responsive microtissues, such as endothelial-lined vasculature.

In a novel study, Balakrishnan et al. [103] created a gelatin-based hydrogel blended with alginate, and cross-linked in the presence of 0.1 M borax for cartilage tissue regeneration. As a result, primary mouse chondrocytes within the designed hydrogel displayed adhesion, viability, and proliferation. Moreover, the results indicated that a self-cross-linked oxidized gelatin-based hydrogel might be a potential injectable matrix for neo-cartilage growth in osteoarthritis treatment. Nevertheless, further progress is needed before this adhesive hydrogel can be used to treat and control initial osteoarthritis when defects are minor and generally linked with a poor healing process.

Functional thermosensitive biomaterials have been introduced and designed as contemporary prospective therapeutic candidates for TE applications. Accordingly, Satapathy and colleagues [104] created a cationic photothermal triggerable-guidable gelatin-based hydrogel comprising polyethyleneimine (PEI)–polypyrrole (Ppy) conductive nanoparticles with a porous structure and observed positive interaction of L929 fibroblast cells with test specimens. In addition, an in vivo investigation was conducted on Wistar rats with a full-thickness wound, to assess the safety and effectiveness of the proposed gelatin–PEI–Ppy hydrogel in the healing process, which revealed no evidence of inflammation or cytotoxicity in either the experimental or control groups. Additionally, the gelatin-based hydrogel scaffold used in their design compensated for the toxicities of the PEI–Ppy at the injured area, allowing for full-thickness skin wound repair. Other possibilities for the developed hydrogel include antibacterial properties, the creation of free radicals to aid tissue repair, and the modification of the potential photothermal therapy for skin TE (Figure 3B).

Natural hydrogels that are often used for promoting epidermal healing are now mostly collagen- and gelatin-based materials, which replicate the native skin ECM, but have poor and unpredictable mechanical and degrading features. In this case, Zhao et al. [105] developed a GelMA-based hydrogel with customizable mechanical, biodegradation, and biocompatibility properties for skin regeneration. This hydrogel was cross-linked under UV exposure in the presence of 0.5% *w*/*v* Irgacure 2959 as a photoinitiator. The results indicated that changing the hydrogel concentration could easily increase the mechanical strength and decrease the degradation features of the produced hydrogels. Furthermore, all concentrations of hydrogel demonstrated superior cell survival (>90%), with increased cell attachment and proliferation associated with higher concentrations. The hydrogels also aided keratinocytes to proliferate and differentiate. GelMA hydrogels’ resistance and programmable features demonstrated that the keratinocyte-laden hydrogels can be employed as epidermal replacements or scaffolds for various in vitro skin treatments.

### 3.3. Serum Albumin-Based Hydrogels 

Serum albumin (SA), the most abundant protein in mammalian blood plasma, is the principal transporter of various solutes within plasma [175,176,177]. Generally, SA responds to pH and temperature changes, is soluble at high concentrations, and gels quickly under certain circumstances [178,179].

Zhou and colleagues [106] adjusted pH value (with NaH_2_PO_4_ and Na_2_HPO_4_) and used PEG-disuccinimidyl succinate (SS_2_) as a cross-linker to generate a human SA (HSA) hydrogel. In addition, bioglass was mixed into the gel to increase its gelation period and facilitate the release of calcium and silicon ions to the injury site during the injection of the acellular hydrogel. They evaluated the epidermal and dermal thickness as well as the angiogenesis; interestingly, they observed that acellular HSA-PEG-SS_2_ hydrogels considerably enhanced wound healing. 

The mechanical properties of a hydrogel have been found to be proportional to its cross-linking density, stiffness, porosity, and concentration. The hydrogel scaffolds’ mechanical features affect cell migration, propagation, and differentiation in three dimensions. Furthermore, the hydrogel’s mechanical stability enhances the scaffold’s structural capacity in a way that it can withstand tensile and pressure loads from the surrounding tissue [180]. In this regard, Liu et al. [107] studied the injectable bovine SA (BSA)-based hydrogel, which was initially produced for the therapy of bone defect repair using thiolated BSA (sBSA) and the strong–silver monosulfide (SAg) coordination, that showed self-healing potential, as well as antibacterial features. Firstly, the BSA was modified with Traut’s reagent; in essence, the lysine residue’s primary amine group was substituted with the thiol group in its primary structure. Thereafter, the sBSA was cross-linked with silver nitrate. Once sBSA and silver were combined, SAg coordination occurred rapidly, resulting in a shear-thinning gel. The mechanical characteristics of the hydrogels can also be altered by changing the quantity of BSA. In this experiment, by changing the BSA ratio in the hydrogel, its modulus could be modified. Furthermore, this unique PBH was degradable, and could gradually release silver ions for creating an antibacterial action against the bacteria *P. gingivalis* and *F. nucleatum.* Additionally, the injectable BSA-based hydrogel promoted bone repair (nearly six-fold) compared to the control group, and showed excellent biocompatibility and bioactivity. In vitro investigations revealed that the hydrogel significantly increased the osteogenesis differentiation of osteogenic precursor cells (60%) after fourteen days. Compared to commercial spongious bone replacements, the in vivo report showed faster and superior bone regeneration in a rabbit model with a major cranial lesion. As a result, this SA-based hydrogel is an excellent candidate for bone repair.

Yuan and a group of researchers [108] developed a dual-network sodium alginate-BSA with hydroxyapatite compound (S-B-H) hydrogel structure for cartilage regeneration, which was cross-linked with CaCl_2_. The S-B-H hydrogel structure showed good physical features, such as outstanding mechanical toughness, high porosity, and water sorption, along with great biological properties that led to the enhancement of viability (up to 40%), differentiation, and proliferation of human bone marrow-derived mesenchymal stem cells. In addition, the in vivo investigation revealed that the S-B-H hydrogel structure could clearly support the development of new cartilage that merged well with native tissues, and was comparable in thickness to nearby cartilage tissue. Hence, the S-B-H hydrogel structure was regarded to have a large potential in treating cartilage injuries.

**Figure 4:**
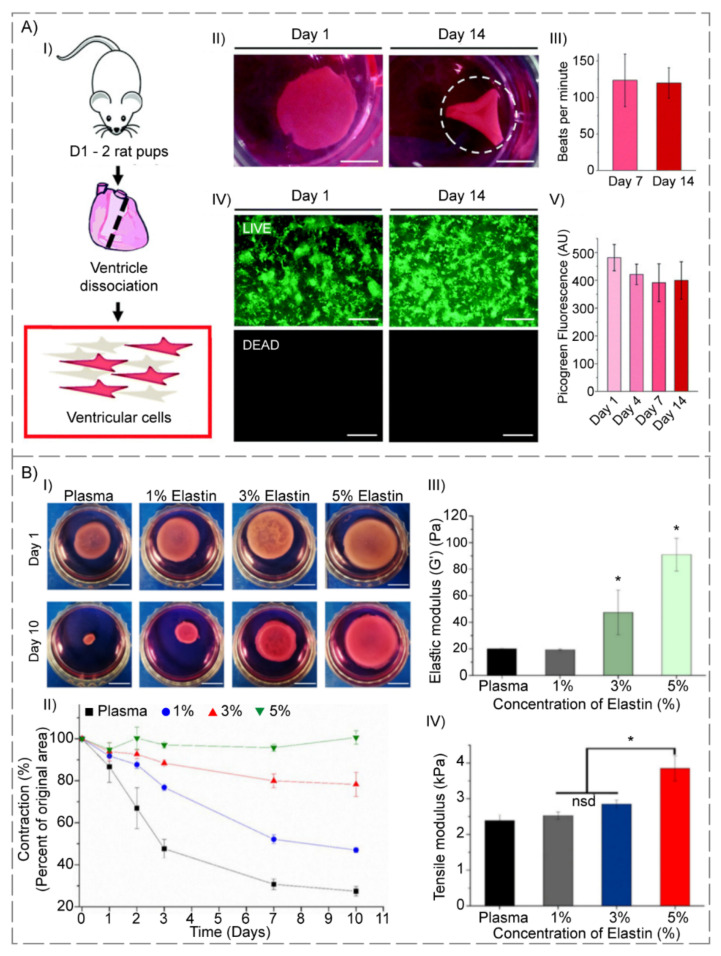
(**A**) Ventricular cells on BSA substrates: (**I**) Diagram of ventricular cell isolation. (**II**) Full substrate images at days one and fourteen, showing the progressive folding of the hydrogel (dashed line represents the estimated original size of the sample). (**III**) Macroscopic beating rate comparisons at days seven and fourteen, suggesting a stable function (*p* > 0.05). (**IV**) LIVE/DEADTM staining of gels seeded with 500 k ventricular cells at days one and fourteen. (V) Picogreen dsDNA quantification of constructs with no difference between time points (*p* > 0.05). Scale bars: (**II**) 5 mm; (**IV**) 500 µm (reproduced content is open access) [109]. (**B**) (**I**) Images of each gel composition in culture medium at time points one and ten days. Percentages indicate % of elastin in elastin-plasma hydrogels. The scale bars correspond to one cm. (**II**) Cell-induced hydrogel contraction ratio for each hydrogel composition (n = 3); Data reported as mean ± SD. Percentages indicate % *w*/*v* elastin content in plasma hydrogels. (**III**) Elastic modulus (G’) obtained from strain sweep tests of plasma and hybrid plasma-elastin hydrogels. (**IV**) Tensile modulus obtained from strain sweep test of plasma and hybrid plasma-elastin hydrogels (* *p* < 0.05) (reproduced content is open access) [111].

Amdursky et al. [109] observed that neonatal rat ventricular cardiomyocytes (NRVCMs) cultivated on a cross-linked BSA hydrogel (incubated at 75–80 °C) that was modified with fibronectin, resulted in NRVCMs with gene profiles, namely connexin 45, connexin 43, ryanodine receptor 2, phospholamban, membrane sodium–calcium, calcium homeostasis, muscle actin, cardiac troponin T, cardiac a-actinin, myosin light and heavy chains, similar to the freshly extracted cardiomyocytes, while NRVCMs that grew merely on the glass began to de-differentiate. Moreover, co-culturing NRVCMs on the substrate with mouse endothelial, muscle and fibroblast cells led to the formation of a hydrogel contractile heart tissue that could be controlled by external electrical stimuli (Figure 4A).

### 3.4. Elastin-Based Hydrogels 

Elastin, one of the main elastomeric proteins in ECM, is present in the elastic fibers of the connective tissue [181], skin [111], cartilage [182], vessel [183], lung [184], ligament, and tendon [185], providing them with elasticity properties. Elastin comprises about 800 amino acid residues, such as glycine, valine, alanine, proline, and lysine [186]. 

It can be a reliable and efficient biomaterial for engineering elastic tissues due to its unique physical and biological properties, including structural stability, elastic resilience, bioactivity, and self-assembly ability [187]. 

Wang and colleagues [110] created a 3D collagen–elastin-based hydrogel using 1 M sodium hydroxide and incubated the structure at 37 °C. The final hydrogel could mimic the natural ECM of the aortic valve due to the combined elasticity and strength of collagen layers. To produce an in vitro 3D co-culture of cardio cells, heart valve interstitial cells (VICs) were encapsulated in the collagen–elastin-based hydrogels, and heart valve endothelial cells (VECs) were cultivated onto the surfaces. VICs consistently expressed integrins β1 (CD29) and F-actin over a seven-day interval, proliferated continuously, and the cell growth was twice on day five; however, cell shape changed to more extended forms. VECs retained their endothelial phenotype until day five, as evidenced by limited F-actin and CD29 expression, and converted VECs made up around 7% of the overall VECs in cultures. Almost 20% of VECs converted to a mesenchymal phenotype on day seven, as evidenced by an increase in F-actin and CD29 expression. These results showed that their collagen–elastin-based hydrogel constructs provide novel scaffolds for studying cell–cell and cell–matrix interactions in vitro, and can be suitable candidates for heart valve TE.

Stojic et al. [111] embedded an elastin-like recombinant (ELR) double cross-linked structure into the plasma-derived fibrin hydrogels to improve mechanical features and bioactivity. The structure was divided into two kinds of ELR, one adapted with azide (SKS-N3), and another with a cyclooctyne (SKS-Cyclo) chemical group at three distinct SKS (serine-lysine-serine). Once SKS contents were equal to or greater than 3%, their findings indicated a reduction in gelation interval and contractions in both the presence and absence of encapsulated human fibroblasts (HFBs), stronger mechanical characteristics, and an improvement in elasticity. This was due to the creation of a complete interpenetrating polymer network comprising both a fibrin network and another network created through reaction amongst azide- and cyclooctyne-modified ELRs. Moreover, as SKS content was increased, a compact interior morphology was revealed, which could be responsible for mechanical enhancement. Nevertheless, HFBs’ proliferation improved once the least SKS contents (1 wt.%) were utilized, but began to decrease whenever the SKS concentration was increased after two weeks compared to the control. As a result, elastin hybrid-plasma hydrogels plus an SKS concentration of 1 to 3% appeared as promising candidates for employment in wound dressing. These positive results demonstrated the importance of finding the right balance involving decreased contraction, improved mechanical characteristics, and biological capabilities, and they pointed to the possibility of using this kind of elastin hybrid-plasma hydrogel as a platform for pharmaceutical goods.

Pal and co-workers [112] developed recombinant human bone morphogenetic protein-2 (rhBMP-2) and doxycycline-loaded collagen-ELP-based hydrogels with biomechanical features that were greater in collagen–ELP hydrogels for osteogenesis. Whereas all hydrogels exhibited 3D interconnected pores (ranging from 160 to 400 µm), the collagen–ELP hydrogels showed a significantly greater modulus of 35 ± 5 kPa than collagen ones. This architecture facilitated human adipose-derived stem cells’ (hASCs) adhesion and proliferation from the first days of cell culture, generating a dense cellular sheath after twenty-one days. The attachment, propagation, and differentiation of hASCs were all promoted by all hydrogels. Furthermore, *E. coli*, *S. sanguinis*, and *P. aeruginosa* displayed biocompatibility against all drug-loaded hydrogels. As a result, collagen–ELP hydrogels have the potential to tackle bacterial infection, while also facilitating directed bone healing.

Staubli and a group of researchers [113] proposed changing the cell adhesion features of elastin-based hydrogels to control the stromal vascular fraction (SVF) cells’ angiogenic capability. Human SVFCs, encapsulated in RGD-REDV-bioactivated or unchanged elastin-based hydrogels, were transplanted in the rat subcutaneous, either directly, or after a five-day incubation. Because of the PBHs’ ability to finely manage the angiogenic and inflammation procedures at the recipient’s location, elastin-based hydrogels have a lot of potential for determining the effective integration of engineered replacements (Figure 4B).

### 3.5. Keratin-Based Hydrogels 

Keratin is a fibrous structural, cysteine-rich, and insoluble protein that can be categorized into two types: soft and hard keratin. Specifically, soft keratin is present in epidermal keratin, such as the stratum corneum in the epidermis, whereas hard keratin is located in the hair, nails, horns, and feathers. More importantly, hard keratin has greater mechanical strength compared to the soft one, because of possessing a higher cysteine concentration [188].

As a vital component of animals’ hard tissues, keratin contributes to tissue tolerance against external forces. In fact, inter- and intra-molecular disulfide linkages in the keratin’s chemical structure are critical for its high mechanical strength [188]. This protein is a qualified candidate for the TE field, due to its biocompatibility, non-immunogenicity, and excellent cellular interactions between primary amino acid sequences and cellular integrins, such as RGD, glutamic acid-aspartic acid-serine (EDS), and leucine-aspartic acid-valine (LDV), to name but a few [189]. 

Since animals do not typically contain keratinase or other enzymes to degrade keratin in their bodies, the in vivo breakdown mediated by proteolytic enzymes for ordinary proteins does not apply to keratin. Thus, keratin is comparatively stable in vivo, in comparison with other fibrous proteins. Moreover, keratin-based hydrogels can withstand enzymatic degradation for a longer time than the others. In particular, keratin solution should be cross-linked by an active ingredient or via stimulating the development of disulfide bridges in order to create the hydrogel [190]. In this regard, cross-linkers include glutaraldehyde [191], transglutaminase [192], dialdehyde [193], ethylene glycol diglycidyl ether [194], glyoxal [195], and formaldehyde [196]. In addition, oxidized cellulose nanocrystal serves as both a reinforcing agent and a keratin cross-linker. It is also noteworthy to mention that thiol functional groups of cysteine within keratin assist its surface modification, with a wide range of chemical components for hydrogel synthesis [197].

Xu and co-workers [114] developed a keratin-based hydrogel utilizing extensively cross-linked keratin isolated from feathers, being entirely detached and decross-linked into linear and oriented molecules with retained molecular weight. The solvent was successfully deposited into scaffolds, with tiny protein strands organized irregularly in different dimensions. Consequently, keratin hydrogels exhibited natural stability due to their disulfide cross-linked molecular chains, and cultivated adipose-derived mesenchymal stem cells easily proliferated and chondrogenically differentiated, suggesting cartilage TE potential for this novel hydrogel.

Veerasubramanian et al. [115] mixed Avena sativa (Oat) with keratin derived from human hair, and the keratin-based hydrogel cross-linked by sodium trimetaphosphate was implanted in rats possessing mock diabetic wounds with induced hyperglycemia, in order to investigate the consequences over twenty-four days. After the mentioned period, keratin hydrogels improved wound healing compared to the normal dressing controls. An in vitro degradation experiment revealed that scaffolds lost their mass (about 65%) after five weeks, and the mouse embryonic fibroblast cells (NIH3T3) cell viability increased to approximately 90% of the control group after forty-eight hours. Furthermore, these hydrogel-based scaffolds demonstrated biocompatibility, antioxidant activity, collagen formation, antibacterial property, and rapid wound repair in diabetic rats. Histological investigations illustrated the development of an epidermis layer and blood vessels in this experiment.

In novel research, Chen and his colleagues [116] developed a disulfide shuffling approach for preparing keratin-based hydrogels via oxygen (O_2_) oxidation. A glucose-triggered in-situ keratin-based hydrogel was produced following this disulfide shuffling technique, using a greater oxidation intensity of hydrogen peroxide (H_2_O_2_). The hydrogel solution contained keratin, cysteine, and glucose oxidase. Furthermore, the keratin-based hydrogel obtained in situ gel formation on the full-thickness wound in rats within three minutes, which can be used as a substrate for wound healing in diabetic rats, particularly by advancing the angiogenesis and vascularization in ulcers.

In another experiment, Cao and a group of scholars [117] created a moderate and plain disulfide shuffling method to produce keratin-based hydrogels inspired by the abundant concentration of intra-molecular disulfide bonds in keratin protein. The natural intra-molecular disulfide bonds were already broken with a reductive reagent, like cysteine, to release the free thiol group, which could then be oxidized to produce inter-molecular disulfide bonds. Furthermore, the inter-molecular disulfide bond density was adjusted by controlling the cysteine content, resulting in a programmable in vivo degradation, controllable disulfide cross-linking concentration, and controllable release rate. Additionally, by eliminating the additional chemical cross-linkers during the production process, this technique ensured that the material was biocompatible. They believed that this research would pave the way for developing PBHs with superior biocompatibility and customizable capabilities, which could be useful in TE applications such as bone and wound healing.

### 3.6. Resilin-Based Hydrogels 

Resilin, one of the most flexible rubber-like proteins, is an insoluble and heat-stable [198] biomaterial with adequate elasticity, low stiffness, high elongation, outstanding resilience, efficient energy storage, and an excellent fatigue life-cycle that can survive numerous contraction/extension cycles, more than 400 million times [198]. Moreover, resilin’s strong resilience permits its quick return to the former shape after being extended for weeks [199]. It has been challenging to create the primary sequence of resilin out of its natural origin, owing to its low stability during the purification process. The product of the CG15920 drosophila gene, as a precursor of resilin with 620 amino acid sequence, has three exons, with amino- and carboxyl ends in each unit [200,201].

Within an intriguing study, Renner and colleagues [118] employed a cross-linker named tris(hydroxymethyl)phosphine for the formation of PBH composed of resilin motif’s repetitive sequences obtained from an insect and cell-binding domains achieved from fibronectin. Specifically, the addition of a bioactive domain to resilin sequences did not affect the protein’s secondary structure. Furthermore, Young’s modulus in the compression condition was 2.4 MPa, being comparable to that of living cartilage tissue, and human MSCs cultivated on the resilin-based hydrogel showed a 95% confidence viability in a three-day time period, and could interact with the cell-binding regions in a sequence-specific method. Therefore, this resilin-based hydrogel might be of use for cartilage bioengineering. 

McGann et al. [119] reported the synthesis of three resilin-based hydrogels offering combined benefits of improved structural, functional, and biological properties over their individual components. Briefly, high-molecular weight resilin-like polypeptides were cross-linked into cysteine residues using a PEG-vinyl sulfone cross-linker, and aortic cells were encapsulated into them. During the seven days of incubation, the encapsulated fibroblasts remained viable, and obtained a spread form analogous to the native fibroblast cells. As a result, these hydrogels have great potential for cardiovascular applications.

In another experiment, Li and a group of researchers [120] created a number of resilin-inspired rubbery hydrogels, with mechanical properties comparable to those used in vocal fold tissue, as well as potential in vitro cytocompatibility and in vivo biological properties. They conducted experiments in which resilin-based hydrogel was injected into the hypodermic tissue of rats. The quick reactions of chemical cross-linking by tris(hydroxymethyl phosphine) allowed for easy injection and guaranteed a rapid transition of the viscoelastic resilin solution to a strong hydrogel in the hypodermic area in vivo. The hydrogels displayed shear moduli in the 1 to 2 kPa range. Three weeks following injection, histological stains and gene expression profiles revealed minor inflammatory profiles, suggesting the potential applicability of these elastomer-like materials for in vivo injection. Using cytocompatible chemistry, resilin-like polypeptides can be coupled with some other synthetic materials to increase flexibility and bioactivity for further in vivo injection of the hydrogel into vocal fold muscles.

Su and co-workers [121] attempted to modify a resilin-like protein named RZ10-RGD; therefore, they used a cleavable cross-linker (3,3′-dithiobis(sulfosuccinimidyl propionate)) with a disulfide bond to create redox-responsive smart resilin-based hydrogels. These hydrogels had a porous microstructure and an elastic modulus of nearly 3 kPa. Furthermore, the survival of NIH/3T3 fibroblasts grown on resilin-based hydrogels for one day was remarkable (>95%). These results showed that smart resilin-based hydrogels have the potential to be used in a wide range of applications, such as TE and drug delivery, that tackle tumors’ intracellular reductive conditions.

### 3.7. Silk-Based Hydrogels

Silk, an attractive structural protein, is made by insects, spiders, scorpions, mites, fleas, and worms, and is made up of a core protein, fibroin fiber, and a glue-like covering comprised of the sericin protein [202]. 

Hydrophobic interactions can physically cross-link the silk-based hydrogels through intra-and inter-molecular β-sheets. Indeed, the formation of a β-sheet structure within silk improves its poor gelation and solubility in water. Increasing the β-sheet structure can be enabled via a change in pH, or by applying shear stresses through using ultrasonication or vortex mixing [203]. In this regard, the sonication modifies the hydrophobic interface and facilitates the self-assembly of silk fibroin molecules [204]. Silk-based hydrogels are generated through a sol-gel transition in the presence of acid, ions, or other additives [205]. The other parameters influencing the gelation reaction are the concentration of fibroin, molar ratio of additives such as Ca^2+^, KCl, glycerol, and poly-(ethylene oxide), and employed fabrication techniques, like shearing, high energy ultrasonication, osmotic stresses for bulk water removal, heating, exposure to solvents, gases and surfactants such as sodium dodecyl sulfate and a sodium N-lauroyl sarcosinate [206].

Being lightweight, fairly strong, flexible, and possessing mechanical features even higher than some polymers like Kevlar [207], silk protein has been widely researched for various biological applications, due to its suitable biocompatibility, minimum thrombogenicity, slow degradability, and low immunological reactivity. Additionally, it is the most versatile protein amongst the natural proteins employed for hydrogel formation [208], and surface treatment approaches are able to extend its applications. Besides, this protein can promote stem cell attachment, proliferation, and differentiation in situ, and improve tissue healing in vivo [209].

In a novel experiment, Patra and co-workers [122] studied the capacity of Antheraeamylitta silk fibroin in cardiac TE. Silk fibroin hydrogel promoted three-day rat cardiomyocytes’ attachment, metabolic activities, sensitivity to extracellular signals, and cell–cell interactions. In addition, they observed that Antheraeamylitta silk fibroin has properties similar to fibronectin, a naturally occurring element in cardiomyocytes, implying that this silk-based hydrogel is an appropriate option for cardiac repair.

Furthermore, Moses and a group of scholars [123] developed a composite nanoapatite/silk-based MSCs-laden hydrogel employing enzyme-mediated cross-linking using extrusion 3D-bioprinting for cartilage and bone regeneration, that was capable of reproducing the osteochondral structure. Of note, nanoapatite particles were used to help mimic the tissue nature. The hydrogels facilitated the creation of an undulating demarcation region at the boundary of chondral and bone sections, in addition to allowing spatial development and the osteogenic and chondrogenic differentiation of encapsulated MSCs.

Hong and colleagues [124] used glycidyl-methacrylate (GMA) to 3D-print silk fibroin-based hydrogel and cross-linked the hydrogel under UV exposure in the presence of 0.6% *w*/*v* lithium phenyl-2,4,6-trimethylbenzoylphosphinate (LAP) as an initiator. The capacity of rabbit chondrocyte-loaded silk-GMA to produce chondrogenesis in in vitro culture was assessed, and then the structures were implanted in white rabbits. The viability, propagation, and differentiation of chondrocytes could be ensured after four weeks of in vitro culture. They revealed that proliferation was increased three times compared to the control group, and demonstrated that human chondrocytes proliferated excellently in 30% silk-based hydrogel during fourteen days of culture. In addition, in vivo studies using a rabbit model with a partly damaged trachea revealed the presence of a new cartilage-like structure and epithelium around the silk-GMA hydrogel. This research suggested that a fabricated silk-GMA hydrogel might be used in TE applications requiring mechanical qualities, such as cartilage regeneration.

In another investigation, Pankongadisak and Suwantong [125] used chitosan and silk fibroin to make injectable hydrogels at physiological temperatures. To promote osteoblast development and increase mineralization in bone cells, the crude water extract of longan seed (WLS) was loaded in this hydrogel. The major interaction between the amino group of chitosan and the phosphate group of β-glycerophosphate disodium (BGP) resulted in the development of hydrogels at physiological temperatures. Furthermore, the increased connection between surrounding macromolecular chains induced by the silk fibroin molecules led to a reduction in the gelation period. At 37 °C, these hydrogels took 4–7 min to change from sol to gel. Besides, these hydrogels were non-cytotoxic toward both NCTC clone 929 and MC3T3-E1 (mouse fibroblast and osteoblast, respectively) cell lines, and positively impacted MC3T3-E1 cell adhesion and proliferation on the hydrogels. The results showed that cell viability in the hydrogels was 60% more than the control group. These hydrogels also demonstrated antibacterial action against *E. coli* and *S. aureus*. Consequently, the hydrogels might be useful as drug carrier systems in bone regeneration, particularly for healing alveolar bone injuries.

## 4. Delivery Strategies 

### 4.1. Infused GF Delivery

**Figure 5:**
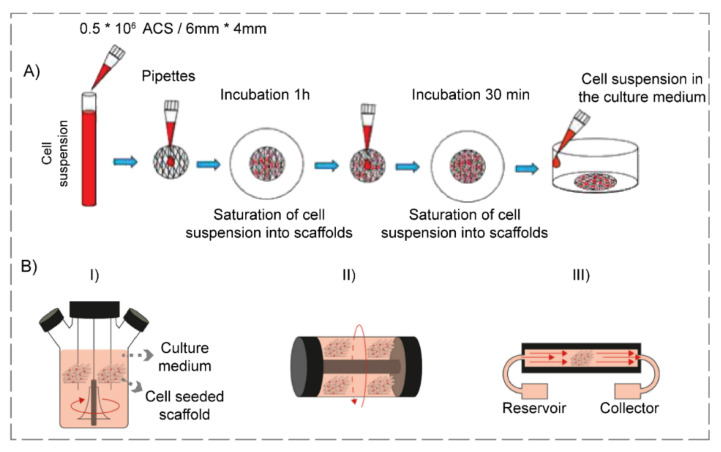
Schematic representation of infused GF delivery, containing (**A**) Static seeding and (reproduced content is open access) [211] (2019, Hindawi) and (**B**) Bioreactor utilization including (**I**) spinner flask, (**II**) rotating wall vessel, and (**III**) perfusion (reproduced content is open access) [212] (2018, BMC).

Infused delivery of GF is a technique in which GF’s penetration within the hydrogel is possible via the diffusion process. Combining GFs with cell culture media and their direct delivery to the hydrogel is accomplished by manual or mechanical methods, which are static scaffold seeding (Figure 5A) and bioreactor utilization (Figure 5B), respectively [210].

Scientists have investigated the delivery of various GFs employing static scaffold seeding throughout the years. Concerning this subject, Xu et al. [213] designed an injectable chitosan-hyaluronic acid scaffold embedded with nerve GF (NGF), since combining the advantages of chitosan and hyaluronic acid could provide a much better biomimetic microenvironment for the NGF delivery in peripheral nerve regeneration. Briefly, the chitosan-hyaluronic acid hydrogels were prepared via covalent cross-linking technique and were then mixed with NGF solution. The results illustrated that the sustained release of NGF was enabled, and its bioactivity was favorably maintained. After a five-day incubation with rat Schwann cells, the OD value at 450 nm for NGF-loaded hydrogel was 2.5 compared to the control group possessing a value of 1.75, indicating the cellular proliferation and hydrogel’s non-toxicity. Furthermore, high cellular attachment, spreading, and the promoted differentiation of seeded cells were other observations in this investigation. In another innovative experiment, a group of researchers [214] fabricated cell-laden keratin-based hydrogels loaded with insulin-like GF-1 (IGF-1) and basic fibroblast GF (bFGF) for skeletal muscle regeneration. At first, GF solutions were combined with keratin powders in order to produce a 7% *w*/*v* hydrogel, and keratose was added to muscle progenitor cell suspension to fabricate a 15% *w*/*v* hydrogel. Both hydrogels were mixed at a 70:30 keratose–keratin ratio via coupled syringes in the second step. Subsequently, the manufactured scaffolds were implanted in the murine models possessing volumetric muscle loss. The outcomes demonstrated a higher recovery of contractile force (maximum of 180 mN) compared to the control group (maximum of 140 mN), neo-muscle formation, and favorable tissue remodeling [215,216,217]. Besides, they suggested the optimization of the keratin formulation for GF delivery for future evaluations.

An enhanced technique for GF delivery is the utilization of bioreactors. In this regard, perfusion systems, spinner flask, and rotating walls are various types of bioreactors utilized for TE purposes [218,219]. Several experiments have been conducted via adopting this procedure. Regarding this matter, a team of scientists [220] engineered poly(ethylene glycol) diacrylate (PEGDA) hydrogels seeded with GFs in a perfusion bioreactor, and investigated the results to determine the scaffolds’ potential application for liver repair. In summary, PEGDA hydrogels were fabricated, and the scaffolds were seeded with epidermal GF (EGF) and primary hepatocyte cells. Performing various tests, researchers concluded that the cellular viability was maintained, and the albumin production, a particular liver function, was enhanced in the bioreactor (42 μg/mg protein/day) compared to the 2D culture (20 μg/mg protein/day) during a seven-day incubation. In an attempt to utilize a rotary bioreactor for cartilage regeneration, Zhu and co-workers [221] proposed a novel transforming GF β_2_ (TGF-β_2_)-seeded chitosan–gelatin hydrogels. The hybrid hydrogels were prepared, and human ASCs were seeded on them, followed by the dynamic seeding of TGF-β_2_ within the rotatory wall vessel. Furthermore, some of the fabricated GF-seeded scaffolds were cultured in static conditions, in order to compare dynamic and static culture conditions. It was revealed that the mass transfer efficiency of the rotary bioreactor was quicker in obtaining an ultimate equilibrium in comparison with the culture performed in the static condition. Besides, cells cultured within the bioreactor expanded nearly three-fold more than the static state over ten days, and their distribution was more uniform. Cell viability for dynamic culture was 92% in comparison with the static one, being 82% in a ten-day culture period. Eventually, the improved TGF-β_2′_s mass transfer efficiency led to enhanced cellular proliferation and the chondrogenic differentiation of stem cells.

### 4.2. Scaffold Immobilization

Scaffold immobilization refers to the GF incorporation into the hydrogel so as to keep it from unfavorable states, such as exposure to high temperature [222]. This technique is classified into physical and chemical immobilization.

Physical immobilization, which is embedding GFs within the hydrogel, is one of the simplest techniques for GF delivery [223]. This technique is obtained through the layer-by-layer method, surface adsorption, and physical encapsulation [224]. In this regard, Kobayashi and co-workers [225] developed a bFGF-loaded gelatin hydrogel by simply adding ten µg of GF to ten mg of gelatin. Thereafter, the hydrogels were injected into beagle dogs with injured vocal folds to test their efficiency. In essence, they revealed that a single injection of GF-loaded hydrogel possessed stronger regenerative effects on acute vocal fold scarring compared to the injection of bFGF solution. GF’s gradual and controlled release led to enhanced mucosal vibration, approximately two-fold more than the sham group, and improved elastin and hyaluronic acid levels in vocal folds, making this GF-loaded scaffold a promising candidate for acute vocal fold scar repair. Another recent study [226] examined the effects of vascular endothelial GF (VEGF) release from a hybrid hydrogel for chronic wound healing. The aldehyde chitosan-amino-end PEG hydrogel with self-healing property was loaded with VEGF, and was injected into mouse models with wounded tissues. The researchers found that controlled release of GF promoted collagen expression (1.3 times more than the control group), angiogenesis, reduction of inflammatory reaction, and mouse fibroblasts ratio increase in the wound region.

The chemical conjugation method is the activation of the hydrogel’s surface with functional groups and its conjugation with GFs via suitable chemical reactions [227]. In this process, the binding affinity describes how firmly the GF binds to the structure [228]. Besides, the hydrolytic or enzymatic cleavage of chemical bond grafting the GF to the hydrogel controls the GF’s desorption rate, enabling several GF release models, including pulsatile, linear, and sequential release profiles [224]. This technique offers sustained and localized GF delivery and prolonged GF availability; nonetheless, the loss of GF bioactivity during the immobilization process due to harm or the considerable modification of biomolecule conformation can happen [229,230]. Several approaches exist for the chemical immobilization of GFs. Carbodiimide coupling is one of the most popular methods for covalently grafting GFs to the hydrogels. In this regard, 1-ethyl-3-(3-dimethylamminopropyl) carbodiimide, also known as EDC, is a mediator in the reaction between amino groups and carboxylic acid groups [231,232]. In this vein, a group of scholars [233] grafted recombinant human epidermal GF (rhEGF) onto sodium carboxymethyl chitosan hydrogel via EDC. Interestingly, the GF-loaded structure possessed more stability against proteases based on the PUMPT theory, that is the biodegradable polymer’s ability to mask the GF during transfer, followed by the degradation of the polymer, GF unmasking, and restoration of its activity. Additionally, the complex successfully maintained GF biological activity (2.4%) in comparison with that of the control group (0.1%) after twenty hours, and could enhance the proliferation of fibroblasts in vitro. Furthermore, when it was tested on diabetic rats’ wound model, an improved in vivo healing rate was observed. Another prevalent method is the mussel-inspired bioconjuction. In fact, researchers have been inspired by the attachment of mussels to various substrates via 3,4- dihydroxy-L-phenylalanine (DOPA) formation, and have utilized polydopamine (PDA), a biocompatible mussel-inspired biopolymer possessing a structure analogous to DOPA, as the GF immobilizer [234,235,236,237]. Regarding this matter, Gan et al. [238] manufactured bilayer nanohydroxyapatite (HA)-GelMA hydrogels immobilized with TGF-β_3_ in the upper layer and bone morphogenetic protein 2 (BMP-2) in the lower layer via PDA, and bone mesenchymal stem cells and chondrocytes were cultured on them to assess their behavior toward cells. The results revealed that stem cells spread well and had osteogenic differentiation in BMP-2-loaded HA-GelMA-PDA scaffolds, and chondrocytes showed enhanced proliferation, as well as increased GAG content (5400 ng/L) in TGF-β_3_-loaded GelMA-PDA structures, compared to that of GelMA hydrogels (3700 ng/L). When the dual-function hydrogels were implanted into rabbit knee joints with full-thickness cartilage defects, the sustained release of GFs due to the presence of PDA resulted in the formation of well-organized cartilage and subchondral bone in defect models, accompanied by the observation of uniformly aligned chondrocytes. 

### 4.3. Spatiotemporally Controlled Delivery

**Figure 6:**
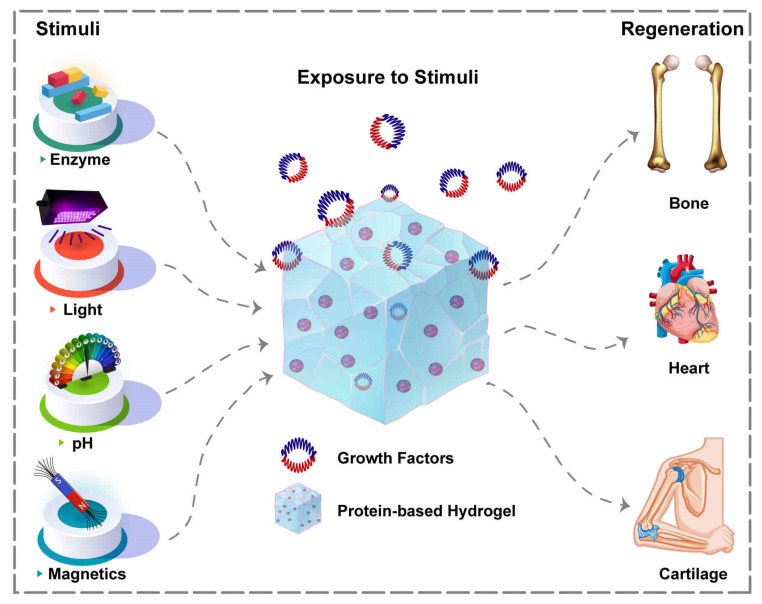
Environmental stimuli playing role in spatiotemporally controlled delivery of GFs from hydrogel systems to various tissues.

The sustained and controlled release of GFs can be achieved by the spatiotemporally controlled delivery method. The release rate of GFs can be modulated via external triggers or biological stimuli, such as pH, enzyme, temperature, and magnetic field, to name but a few (Figure 6) [239]. 

**Figure 7:**
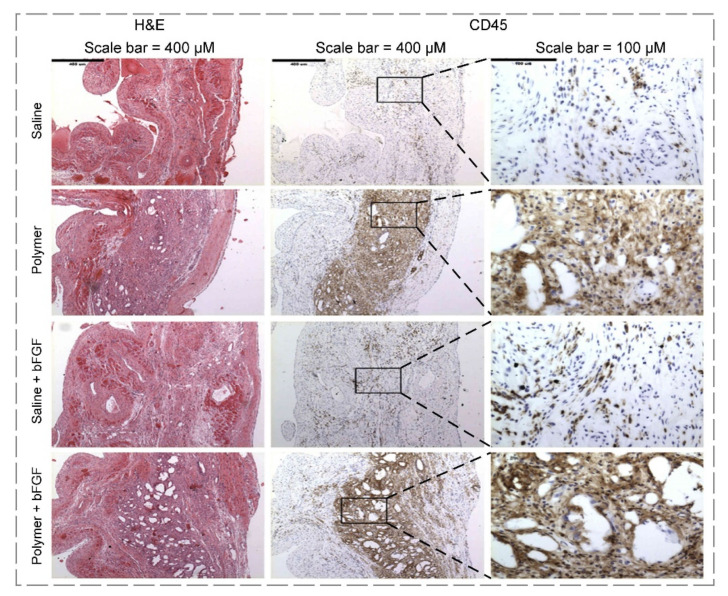
H&E- and CD45-stained images at twenty-eight days after injection with saline, polymer, bFGF+saline, and bFGF+polymer. As shown, an increased inflammatory response was indicated by CD45 staining in animal models injected with polymer, in comparison with saline at twenty-eight days (reproduced content is open access) [242].

A pH-responsive structure refers to a GF-loaded complex that releases GF by material dissolution when the pH of the target tissue changes [240]. A shift in pH usually occurs in tissue injuries, and the system mentioned above utilizes the altered pH of the tissue microenvironment to release GFs. Regarding this matter, Garbern and co-workers [241] designed bFGF-loaded injectable hydrogels for the treatment of infarcted myocardium. As a pH-responsive hydrogel, poly (N-isopropylacrylamide-co-propyl acrylic acid-co-butyl acrylate) transitioned from the liquid form at pH 7.4 to gel form at pH 6.8 in 37 °C, and they took advantage of this property in an acidic condition of male rats’ ischemic myocardium. Conducting various experiments, they deduced that the injection of bFGF-loaded hydrogel to animal models and its sustained release profile resulted in GF’s long-term retention in comparison with the saline+GF complex, increased capillary and arteriolar densities up to 30–40% than polymer-only or saline+bFGF controls, enhanced angiogenesis, and improved cardiovascular function (Figure 7). Nevertheless, one drawback of these systems is that acidic pH is not suitable for all GFs [242]. 

Enzyme-responsive systems are sensitive to the presence of special enzymes and release their cargo, a GF, when confronting these physiological cues. For instance, the upregulation of several proteolytic enzymes, including serine proteases, elastases, collagenases, and matrix metalloproteinases, happens within a chronic wound environment [239]. With respect to this issue, a group of scholars [243] engineered PEG-based hydrogels loaded with hepatocyte endothelial GF (HGF) and VEGF that had programmed release triggered by collagenase exposure. To delineate, PEG-maleimide macromers were functionalized with the RGD peptide, as well as GFs. Subsequently, the precursor solution was cross-linked with a protease-degradable peptide sequence, VPM, to fabricate the final injectable hydrogel. Due to the hydrogel’s collagenase-mediated degradation, the controlled release of GFs was achieved. Furthermore, Sprague–Dawley rats with acute myocardial infarction were treated with these hydrogels, and the results revealed a significant enhancement in angiogenesis (sixteen vessels/mm^2^ in GF-loaded complex and eight vessels/mm^2^ in the group receiving ischemia/reperfusion surgery after twenty-one days), fibrosis prevention, cardiac progenitor cells’ migration, and enhanced cardiac function.

Another method of spatiotemporal delivery is triggered delivery, which is basically the release of GF due to physical alterations initiated by an external stimulus. Two popular examples of this technique are magnetic and light stimuli [239].

The GF-loaded magnetic hydrogels alongside magnetic fields are commonly used in TE to deliver GFs spatiotemporally. The movement of particles toward the injury site is accomplished by applying a magnetic field, and then the bioactive factor is delivered to a particular cell mass [244,245,246]. In an innovative experiment, Kim et al. [247] fabricated magnetic-responsive TGF-β_1_-loaded heparin-modified alginate hydrogels containing iron oxide nanoparticles. The structure’s exposure to the magnetic field (120 cycles for nearly 2 min, every 24 h) was tested, and they concluded that the hydrogel’s deformation could accelerate the GF release. The maximum release of GF in the absence of the magnetic field was 4.6 ng, whereas its release was 1.5 ng in the presence of the magnetic field, showing the positive effect of the magnetic field that led to the slow GF release. Additionally, the chondrogenic differentiation of ATDC5 cells, derived from mouse teratocarcinoma cells, was the consequence of this investigation. Despite the favorable results that these systems can reveal, a disadvantage of employing magnetic particles is that some of them can cause cytotoxic effects [248].

Employing light for the cleavage of molecules is another approach that a group of researchers utilized to release BMP-2 and BMP-7 [249]. Briefly, GFs were conjugated to photocleavable units, and then the complex was bound to PEG hydrogel. By choosing various wavelengths of light, GFs release could be regulated, due to the cleavage of units. Moreover, the BMPs’ bioactivity was affected by neither BMPs’ covalent modification nor light exposure, and the osteogenic differentiation of human MSCs was confirmed due to the elevated levels of alkaline phosphatase, being 2–4 fold higher than the control groups.

GFs can be delivered via different methods, including infused technique, scaffold immobilization, and a spatiotemporally controlled approach to the desired tissues (Table 2). Since hydrogels, as GF delivery systems, offer important advantages, such as GF long-term retention, maintenance of GF bioavailability, and more importantly, sustained release of these proteins, they have been extensively employed in the TE field.

## 5. Critical Determinants of GF and PBHs

All the strategies and methods mentioned above are used to prepare GFs and PBHs as suitable engineered systems. However, reaching the top results in TE can be limited if scholars do not consider several important factors. In this regard, multiple principal parameters, including pH, concentration, stability, bioactivity, clearance rate, bioavailability, and route of administration, should be meticulously considered for enhancing the efficiency of proteins. 

One of the key considerations is pH, since proteins’ stability in various pH values can be different. As an example, fibroblast GF (FGF20) is unstable at pH < 5 [250]. For increasing their stability within the wide range of pH values, GFs are delivered via proper biomaterials such as hydrogels [251]. As mentioned, proteins have ionizable groups such as carboxyls and amines, and since these groups’ charge depends upon the pH value, a protein can have various charges at different pH values [252]. The pI of a protein refers to the pH at which the protein carries no net electrical charge and thus is neutral [253]. Considering the value of pI for each GF is of high importance in designing GF-related experiments. For instance, acidic gelatin (pI of 4.7–5.2) can be employed for the basic GFs’ sustained release, while basic gelatin (pI of 7–9) can be utilized for the acidic GFs’ sustained release [251].

Although GFs are incredibly effective in promoting cellular proliferation and tissues regeneration, the amount of released GF influences this issue exceedingly. To be more specific, GFs function best at doses within a therapeutic window; hence, at high concentrations, they can be toxic or produce harmful side effects, whereas GFs’ low concentrations can be ineffective [254,255]. By employing hydrogels, suitable doses of GFs are released in the target region. 

The stability of GFs is another important factor, since these proteins possess a short half-life, and can be unstable in specific pH values and temperatures [210,256]. Regarding this matter, a group of researchers [257] revealed that EGF’s monomer content was diminished from 33.73 to 5.61%, in response to an accelerated thermal stress of 70 °C. Therefore, enhancing the proteins stability through the selection of the right strategies is among the number-one priorities [258]. 

Bioactivity is defined by achieving particular effects after exposure to a specific substance; these impacts may contain tissue metabolism, uptake, or physiological response [259]. Choosing the proper biomaterial as the engineered delivery system that is able to maintain the GF bioactivity during its release is one of the significant parameters [260]. Recently, the investigation of Xiao et al. [261] demonstrated that sulfobetaine methacrylate hydrogels were more effective than PEG ones in FGF-2 delivery for wound healing, since they could prolong the release of FGF-2 and successfully maintain its bioactivity. Furthermore, the clearance rate of GFs, the rate at which GF is removed or cleared from the whole or part of the body [262], should be noticed, due to the fact that enhancing or reducing the natural clearance rate of GFs from the body may require additional tasks.

Bioavailability is a critical pharmacokinetic factor, referring to the substance proportion administered via any non-vascular route which gains access to the systematic circulation [263]. With the purpose of enhancing GF bioavailability, Elmasry and co-workers [264] developed a computational model to assess how IGF-1 bioavailability affects the homeostasis of the intervertebral disc (IVD). It was revealed that a decrease in IGF-1 bioavailability diminished the IVD anabolism, while its enhancement as a treatment for degenerated IVDs is only advantageous in regions receiving adequate nutritional supply, and increased bioavailability augments tissue degradation in malnourished sites. Eventually, the GFs’ route of administration is of significance, since their stability and side effects highly depend on this factor [255,265].

## 6. Summary, Challenges, and Outlook

There is still a considerable gap in using PBHs for clinical applications, particularly TE, that should be decreased by overcoming the current challenges. To begin with, in the PBHs’ primary form, conformational changes from the third to second structure lead to the increase of the random coil content with unlimited flexibility in the protein structure, which favors the structure for gelation. Hence, the total denaturation of protein for forming hydrogel is not necessary. Indeed, the structural changes from the third to second structure or increased β-sheet content are sufficient in this procedure. 

Although proteins are composed of highly biocompatible long chains, harmful solvents for fabricating PBHs can affect cell viability and cause cytotoxicity [58,83]. Thus, to boost PBHs’ biocompatibility, numerous other combinations are required to be examined to fully assess the inflammatory and immune responses of the PBHs. In this regard, selecting the proper solvent or decreasing the side effect of the toxic solvent can affect the clinical results.

Moreover, imitating natural systems through protein engineering introduces novel proteins or merges various proteins into hydrogel structures with multiple functions. Regarding the employment of coiled coil-based hydrogels for cancer and TE research, one issue that limits their application is the complexity and high expense of the large-scale synthesis. Nonetheless, the growing interest in these systems creates know-how, and promotes the development of facilities and infrastructures for their large-scale production [266]. Another major challenge is the discovery or design of coiled coils, as smart hydrogels for carrying drugs, that are stable under physiological circumstances, but are able to unfold in response to tiny fluctuations in pH, temperature, etc., within the human body [85]. The field of PBHs has not fully developed yet, so the demand is increasing for protein engineering-based solutions to intricate TE problems. 

Additionally, in situ forming hydrogels, existing in the liquid state at ambient temperature, but experiencing a phase transition once injected into the body, can be combined with anti-cancer drugs or protein-based therapeutics, and are ideal candidates for various cancer therapies, since they provide multiple benefits such as having minimal invasiveness, prolonging the drug delivery, increasing the drugs’ bioavailability, diminishing the side effects, and enhancing the patient compliance. Several obstacles of these systems, including immunogenicity, toxic cross-linking agents, and biodegradability under the tumor microenvironment conditions, should be taken into account in future evaluations to achieve optimized clinically applicable systems [267,268]. Specifically, in the case of protein-based in situ hydrogels, although they offer tissue-specific gelation and controlled release of their cargo, the rational design of them can be challenging, as the hydrogel’s structure and characteristics are highly dependent upon the structure of their building blocks [269]. Furthermore, in order to certainly substitute the conventional treatments with the injectable complex, a novelty in the development of design approaches and new architectures is needed [270].

In cell-laden hydrogels, more effort should be made to improve the penetration and perfusion of fluids to enhance the nutrition delivery and metabolites elimination. Furthermore, the long-term cell adhesion and degradation rate of hydrogels in in vivo conditions should be empowered by further investigations.

The regeneration of injured heart tissue after myocardial infarction remains challenging, due to the lack of electrical properties in the large percentage of implanted scaffolds. As a result, conductive nanoparticles such as Fe_2_O_3_ have been incorporated into the scaffolds, such as hydrogel-based ones, in order to increase their electrical characteristics [271,272]. Besides, repairing an infected skeletal defect is still complicated, demanding the need for multifunctional biomaterials with osteogenesis and antibacterial properties for infected bone regeneration. Concerning this subject, it is fascinating to investigate the performance of PBHs incorporating Ag or Au nanoparticles for employment in bone formation [273]. To treat inflammatory bowel diseases, nanoparticles, loaded with an anti-inflammatory tripeptide, can be encapsulated in a hydrogel, so that the challenge of intestinal inflammation will be solved, since the complex degrades in intestinal fluid at pH 5–6 and reduces the inflammation [274]. Despite their suitable biological properties, several hydrogels require nanoparticle additives to perform better in repairing the target tissues. Although nanoparticles are needed nowadays to achieve the desired properties, future studies can focus on developing PBHs with suitable features that are in no need of adding nanoparticles. This matter may increase the hydrogels’ cost-effectiveness, efficiency, and biocompatibility to a great extent.

Last but not least, hydrogel-based protein delivery systems suffer from several drawbacks for the delivery of proteins, such as uneven tissue development, cell detachment, spatial distribution absence, and protein damage, to name but a few. Future experiments should overcome present obstacles to obtain tailorable GFs’ release and fill the gap between pre-clinical and clinical outcomes. In future evaluations, it is indispensable to consider the target tissue with its unique features and PBHs’ properties, and their sensitivity to the target tissue condition.

Overall, in the field of PBHs, understanding the structure of the protein, favorable interactions between proteins, and polymeric matrix within hydrogels, delivery strategies, and the consideration of critical factors such as biocompatibility are incredibly significant for achieving eye-catching in vivo results.

## Figures and Tables

**Table 1:** Summary of TE-related applications of PBHs.

Proteins	Cross-Linking	Hydrogel Components	Effects of Protein Component	Target Tissue	Ref.
**Collagen**	Physical cross-linking Adjustment of pH (50 mM HEPES), employment of CaCl_2_ and thrombin, and UV exposure	-	Showed rapid mouse myoblast cells’ infiltration and micro-vascularization	Heart	[98]
	Thermally cross-linking Incubation for 30 min	-	Formed a lattice pattern for cornea structure	Cornea	[99]
	-	Alginate	Increased chondrocyte cell viability (up to 90%)	Cartilage	[100]
	Thermally cross-linking Incubation at 37 °C	-	Displayed significant osteogenic differentiation	Bone	[101]
**Gelatin**	Physical cross-linking UV exposure, Irgacure 2959 (0.5% *w*/*v*)	GelMA	Produced endothelial cell-responsive tissues	Blood vessel	[102]
	Chemical cross-linking Borax (0.1 M, 30 s)	Alginate	Promoted mouse chondrocytes’ adhesion, viability, and proliferation	Cartilage	[103]
	-	PEI-Ppy	Developed antibacterial properties	Skin	[104]
	Physical cross-linking UV exposure, Irgacure 2959 (0.5% *w*/*v*)	GelMA	Aided keratinocytes’ proliferation and differentiation	Skin	[105]
**Serum albumin**	Chemical cross-linking Adjustment of pH (NaH_2_PO_4_ and Na_2_HPO_4_)	PEG-SS_2_-bioglass	Accelerated the wound healing process	Skin	[106]
	Ionic cross-linking (Ag^+^)	-	Significantly increased osteogenesis differentiation	Bone	[107]
	Ionic cross-linking (CaCl_2_)	Sodium alginate-Hydroxyapatite	Affected the differentiation and proliferation of human bone marrow-derived mesenchymal stem cells	Cartilage	[108]
	Thermally cross-linking Incubation at 75–80 °C	Fibroin	Created contractile heart tissue	Heart	[109]
**Elastin**	Thermally cross-linking incubation at 37 °C	Collagen	Accelerated the heart valve endothelial cells’ growth	Heart	[110]
	Modification of SKS concentration	Plasma	Improved mechanical characteristics and biological capabilities	Skin	[111]
	Chemical cross-linking N-hydroxysuccinimide (NHS)-1-Ethyl-3-(3-dimethylaminopropyl(EDC)	Collagen	Tackled bacterial infection	Bone	[112]
	-	-	Controlled angiogenesis	Blood vessel	[113]
**Keratin**	Disulfide cross-linking	-	Showed rapid penetration, propagation, and differentiation of MSCs	Cartilage	[114]
	Chemical cross-linking Sodium trimetaphosphate	Konjac glucomannan, Oat	Aided collagen formation	Skin	[115]
	Disulfide cross-linking	Glucose-triggered	Decreased gel formation time	Skin	[116]
	Disulfide cross-linking	-	Developed hydrogel biocompatibility	Bone and Skin	[117]
**Resilin**	Chemical cross-linking Tris(hydroxymethyl phosphine)	Fibronectin	Increased human MSCs’ proliferation	Cartilage	[118]
	Chemical cross-linking PEG macromers	PEG-vinyl sulfone	Increased aortic cell viability	Cardiovascular	[119]
	Chemical cross-linking Tris(hydroxymethyl phosphine)	-	Increased hydrogel flexibility and bioactivity	Vocal fold	[120]
	Chemical cross-linking 3,3′-dithiobis(sulfosuccinimidyl propionate)	-	Displayed remarkable NIH/3T3 fibroblasts’ growth in a day (>95%)	-	[121]
**Silk**	-	Fibroin	Improved rat cardiomyocytes cells’ attachment and activities	Heart	[122]
	Enzyme-mediated cross-linking	-	Provided the repair of osteochondral tissue	Bone and Cartilage	[123]
	Physical cross-linking UV exposure, LAP (0.6% *w*/*v*)	Glycidyl methacrylate	Displayed proliferation and viability of chondrocyte cell after four week	Cartilage	[124]
	Thermally cross-linking at physiological temperature	Chitosan	Positively impacted MC3T3-E1 cells’ adhesion and proliferation	Bone	[125]

**Table 2:** Summary of GFs’ delivery techniques and their related experiments.

Hydrogels Composition	Growth Factor	Delivery Method	Tissue	Ref.
Chitosan-Hyaluronic acid	NGF	Static scaffold seeding	Nerve	[211]
Keratin-Keratose	IGF-1 and bFGF	Static scaffold seeding	Skeletal muscle	[212]
PEGDA	EGF	Bioreactor utilization	Liver	[218]
Chitosan–Gelatin	TGF-β_2_	Bioreactor utilization	Cartilage	[219]
Gelatin	bFGF	Physical immobilization	Vocal fold	[225]
Aldehyde chitosan-amino-end PEG	VEGF	Physical immobilization	Skin	[226]
Sodium carboxymethyl chitosan	rhEGF	Chemical immobilization	Skin	[233]
HA-GelMA	TGF-β_3_ and BMP-2	Chemical immobilization	Osteochondral	[238]
Poly (N-isopropylacrylamide-co-propyl acrylic acid-co-butyl acrylate)	bFGF	Spatiotemporally controlled delivery	Heart	[241]
PEG	HGF and VEGF	Spatiotemporally controlled delivery	Heart	[243]
Heparin-modified alginate-iron oxide nanoparticles	TGF-β_1_	Spatiotemporally controlled delivery	Cartilage	[247]
PEG	BMP-2 and BMP-7	Spatiotemporally controlled delivery	Bone	[249]

## Data Availability

Not applicable.
